# Exploring the values, preferences, and information needs of patients with *NKX2-1*-related disorders: A qualitative study protocol

**DOI:** 10.1371/journal.pone.0281573

**Published:** 2023-02-09

**Authors:** Carmen Martín-Gómez, Juan Dario Ortigoza-Escobar, Laia Nou-Fontanet, Juan M. Molina-Linde, Anne-Catherine Bachoud-Lévi, Juliane Léger, Juan Antonio Blasco-Amaro, NKX2-1-Related Disorders Guideline Working Group

**Affiliations:** 1 Health Technology Assessment Area-AETSA, Andalusian Public Foundation for Progress and Health (“Fundación Progreso y Salud”–“FPS”), Seville, Spain; 2 Research Group HUM604: Lifestyle Development in the Life Cycle and Health Promotion, University of Huelva, Huelva, Spain; 3 Department of Child Neurology, Movement Disorders Unit, Institut de Recerca Sant Joan de Déu, Barcelona, Spain; 4 U-703 Centre for Biomedical Research on Rare Diseases (CIBER-ER), Instituto de Salud Carlos III, Barcelona, Spain; 5 European Reference Network for Rare Neurological Diseases (ERN-RND), Tübingen, Germany; 6 Department of Paediatric Neurology, Hospital Sant Joan de Déu, Barcelona, Spain; 7 Assistance Publique-Hôpitaux de Paris, Henri Mondor Hospital, National Center of Reference for Huntington’s Disease, Créteil, France; 8 Département d’Etudes Cognitives, École Normale Supérieure, PSL University, Paris, France; 9 University Paris Est Creteil, INSERM U955, Institut Mondor de Recherche Biomédicale, Equipe NeuroPsychologie Interventionnelle, Creteil, France; 10 Endocrinology-Diabetology Department, Assistance Publique-Hôpitaux de Paris, Robert Debré University Hospital, Reference Center for Growth and Development Endocrine Diseases, Paris, France; 11 Université Paris Cité, NeuroDiderot, Institut National de la Santé et de la Recherche Médicale (INSERM 1141), Paris, France; 12 European Reference Network on Rare Endocrine Conditions (Endo-ERN), Amsterdam, The Netherlands; Institute of Biomedical and Health Research in Malaga (IBIMA), SPAIN

## Abstract

**Background:**

*NKX2-1*-related disorders have a prevalence of 1:500,000 and are therefore considered a rare condition according to the European Commission’s definition. The European Reference Network of Rare Neurological Disorders is developing the first clinical practice guideline on the management of this condition, with the support of the Andalusian Health Technology Assessment Area, Endo-ERN, ERN-Lung and Imegen, within the framework of the ERNs Guidelines programme (DG SANTE/2018/B3/030). Within the scope of this programme, it becomes necessary to explore the patient perspective in order to include it in the ongoing clinical practice guideline and accompanying patient information booklet.

**Methods and analysis:**

This study will use qualitative methods to explore the values, preferences and information needs of patient with NKX2-1-related disorders and their caregivers. Participants will come from a variety of countries throughout Europe. One focus group and four semi-structured interviews will be conducted. Pairs will analyse the data using Grounded Theory. The Andalusian Regional Ministry of Health’s Ethics Coordinating Committee for Biomedical Research (Sevilla, Andalucía, Spain) has approved this study protocol (29/03/2022).

**Discussion:**

This is the first study to explore the values, preferences, and information needs of patients with *NKX2-1*-related disorders. The proposed study’s findings will contribute to the generation of useful knowledge that will provide guidance to improve the care given to patients with the studied condition. While this study will provide valuable insights into the perspectives of patients with *NKX2-1*-related disorders, the findings are unlikely to be generalizable to patients with other conditions.

## Introduction

A disease is considered rare in the European Union if it affects one person in every 2,000 [1]. Due to their low prevalence, scientific and medical knowledge in the field of rare diseases is limited, creating challenges in the search for diagnosis, treatment, and care options. The European Reference Networks (ERNs) were established with the goal of addressing this shortage and providing the best diagnostic and treatment options for patients suffering from rare diseases. These networks link healthcare providers throughout Europe in order to address medical conditions that require highly specialised treatment and a concentration of knowledge and resources. At present, 24 networks participate in the initiative. Through these networks, reliable Clinical Practice Guidelines (CPGs) and tools to support clinical decision-making must be developed to ensure that people with rare diseases and their caregivers receive safer and more efficient care throughout the European Union. CPGs developed for rare diseases must address the challenges associated with a lack of sufficient scientific evidence as most methodological approaches, such as GRADE [2], require this evidence to make recommendations.

In the aforementioned context, the European Commission (EC) issued a tender in 2018 (DG SANTE/2018/B3/030) with two primary objectives: (i) to assist ERNs and their health care providers in developing, evaluating, and implementing CPGs and other clinical decision support tools; and (ii) to contribute to the ERNs’ and their members’ capacity for producing and adhering to high-quality CPGs and tools in their area of specialisation. The tender was won by a consortium coordinated by the Andalusian Public Foundation for Progress and Health (FPS) and comprised two specialised health training institutions (the Andalusian School of Public Health and the Open University of Catalonia) and five health technology assessment agencies that are members of RedETS (the Spanish National Network for Health Technology Assessment), including AETSA (Andalusian Health Technology Assessment Area). One work package of the project aims to methodically assist ERNs in the development, evaluation and adaptation of CPGs. To accomplish this, each of the consortium’s five agencies supports different ERNs. The study presented here is part of this work package and is contextualised within the AETSA’s support for the European Reference Network on Neurological Diseases (ERN-RND) in developing the first CPG for the management of *NKX2-1*-related disorders.

*NXK2-1*-related disorders are considered rare due to their low prevalence (1:500,000) [3]. Choreoathetosis, hypothyroidism and neonatal respiratory distress are all symptoms of this condition (OMIM*600635). Variants in the *NKX2-1* gene (formerly known as *TITF1*) or a deletion in 14q13.3, even if the *NKX2-1* gene is not involved, can cause it [4]. Additional signs and symptoms such as tremor, myoclonus, dystonia, ataxia, sensorineural hearing loss, hypotonia and motor developmental delay, attention deficit and hyperactivity, hypodontia, short stature, and psychiatric, cardiovascular and genitourinary abnormalities may occur in patients with *NKX2-1*-related disorders [5, 6]. The onset and progression of symptoms, as well as their association with other symptoms, are highly variable in this condition. The entire brain-lung-thyroid syndrome is present in half of patients. Chorea typically begins in early childhood or around the age of one year, or in late childhood or adolescence, and progresses into the second decade, when it remains stable or remits. The second most common manifestation is pulmonary disease, which can manifest itself in newborns as respiratory distress syndrome, in young children as interstitial lung disease, or in the elderly as pulmonary fibrosis. In young adults with an *NKX2-1*-related disease, the risk of developing lung cancer is increased. Thyroid dysfunction caused by dysembryogenesis can manifest as primary congenital hypothyroidism [3]. Although numerous reports have been published on adult-onset cancer associated with *NKX2-1*-related disorders, including large cell lung carcinoma, glioblastoma multiforme and papillary thyroid cancer [7–9], there are conflicting recommendations for follow-up for these patients, ranging from no controls [10] to annual lung X-rays or CT scans [3], as well as thyroid palpation. As a result, *NKX2-1*-related disorders affect a variety of organs and systems, and patients with this disorder also face an increased risk of developing cancer.

For this condition to be addressed comprehensively in the CPG, in addition to the topics to which clinicians attach most importance, it is necessary to give a voice to the patients themselves and to the people who accompany them in their processes, such as their caregivers or representatives, in order to identify the key issues for the people affected. To meet this objective, two main recommendations are supported by the “Guideline International Network Public Toolkit: patient and public involvement in guidelines” [11]. The first of these recommended actions is the inclusion of patients and/or patient representatives in the guideline development group which has been addressed (listed in the acknowledgements section). The second recommended action is to use research methods to ask patients, which is what this study is about. Thus, within this collaborative framework, and to comply with the above-mentioned indications, it is necessary to identify patient values, preferences and information needs in order to incorporate them in the development of the CPG and address them in the patient information booklet incorporating the relevant information of the CPG. The CPG will be developed based on the handbook entitled “Methodology for the elaboration of Clinical Practice Guidelines for rare diseases” [12], developed within the framework of the ERNs Guidelines project and based on the GRADE approach. The GRADE approach involves the Evidence-to-Decision framework, which considers patient values and preferences as a criterion for formulating recommendations [13, 14]. The patient document aims to provide high-quality information based on available evidence and best clinical practices in accessible layman’s language [15, 16]. The document will be developed based on the handbook entitled “Methodology for the Elaboration of Patient Information Booklets for rare diseases” [17], which was also developed as part of the ERNs Guidelines project.

## Aim

The overall goal is to explore, using qualitative research methods, the values, preferences and information needs of patients with *NKX2-1*-related disorders, their caregivers, and/or representatives in order to identify elements that:

➢ are relevant from a patient perspective, in addition to those considered important by clinicians, in order to avoid their omission from the CPG;
➢ can be incorporated into the patient information booklet to address the identified information gaps.

## Methods and analysis

### Study design

This is a protocol for a qualitative study that was designed following the Qualitative Protocol Development Tool of the Health Research Authority [18]. Once finalised, the results will be reported in accordance with the Standards for Reporting Qualitative Primary Research [19] and the Consolidated Criteria for Reporting Qualitative Research (COREQ) [20].

Given the exploratory nature of this study, a variety of qualitative methods will be considered to accomplish the objectives. A focus group and four semi-structured interviews will be conducted to ascertain values, preferences, and information needs of the target patients, as well as their caregivers and/or patient representatives in the healthcare process and communication with health professionals. Additionally, their perceptions of different health-related issues and perceived quality of life will be investigated.

### Participants

#### Profiles

The participants must fulfil the following criteria to participate in the study: (i) be over the age of 18; (ii) be capable of giving informed consent; (iii) be able to communicate effectively (orally and in writing) in Spanish, English, French, or German; (iv) be affected by a *NKX2-1*-related disorder, or be a caregiver or representative of the target patients.

#### Sample

The sampling strategy for this study will be based on what is known as theoretical sampling in qualitative research [21]. Thus, individuals who match the aforementioned profile, are current users of the Spanish health system and speak fluent Spanish will initially be chosen. They will participate in the focus group. Then, to determine whether the results obtained with participants from Spain are maintained at European level, individuals matching the above profile, namely health system users in another European country who are fluent in Spanish, English, German or French will be interviewed. By including participants from various European countries who fulfil the abovementioned profile, the data will be more representative on a European level. Given the low prevalence of the condition studied, if participants from outside Europe could be included, they would be interviewed. In this case, any differences between healthcare services would be considered when reporting the results. The choice of available languages is based on the location of ERN-RND and Endo-RND clinicians working on the CPG in question and who will provide access to study participants.

The study will involve a minimum of eight and a maximum of twelve participants, in accordance with the methodology described. The focus group will consist of at least four and up to eight participants. Additionally, four semi-structured individual interviews will be conducted. The participants in the focus group and semi-structured interviews will be different. The number of participants was determined considering that this is a qualitative study and that the condition studied is considered a rare disease by the European Commission [1], given that previous research has indicated a prevalence of 1:500,000 for pathologies associated with the *NKX2-1* gene mutation [3]. Based on the methodological approach proposed by Malterud, Siersma and Guassora [22] for work samples in qualitative studies, this sample would provide sufficient informative power.

#### Recruitment

Clinical staff from ERN-RND and Endo-ERN will screen, pre-select and contact eligible participants from among their own patients and caregivers based on established criteria. They will explain the study’s characteristics and objectives, the audio-video recording of the focus groups/semi-structured interviews, and will request their participation and consent to transfer their contact information to the study’s research staff. Then, the research staff will contact the pre-selected individuals via telephone or email to explain the study in detail and confirm their participation. The following documents will be emailed to the participants: (i) the information sheet; (ii) the informed consent form; and (iii) the confidentiality form. The latter two forms will require signatures and must be submitted to the research staff. An appointment will then be arranged to conduct the focus group or semi-structured interview.

This form of recruitment may prevent selection bias as potential participants are recruited directly from the registers held by clinicians in the European ERN-RND and ENDO-ERN networks. In other words, given that participation requires confirmation of the genetic mutation causing the condition, we would have access to the majority of patients diagnosed with this condition at European level, given that clinical staff from the aforementioned ERNs are involved in the guideline development group and in this study.

#### Study withdrawal

Individuals who participate in the study will be informed that they have the right to withdraw from it at any time, without having to justify their withdrawal, prior to the focus group or interview, or after data collection, prior to the data being analysed in conjunction with the data of other participants. Additionally, they will be informed that they are not required to provide a reason for withdrawing from the study and that their decision will have no impact on the healthcare they will receive or on any other setting.

### Data collection

Both the focus group and the interviews will take place virtually via the Microsoft Teams platform, and audio and video recorders will be used. The rationale for conducting the study virtually is to facilitate the participation of individuals who are in different geographical locations, as well as to avoid any unexpected situation resulting from the current COVID-19 context. Given the European dimension of the project, the study will include a focus group with participants who can converse fluently in Spanish, as well as semi-structured interviews with participants who can converse fluently in Spanish, English, German or French. An interpreter will translate the conversation between the participant and the researcher during the semi-structured interviews in German or French; the interpreter has a background in research and will sign the confidentiality form.

**Fig 1:**
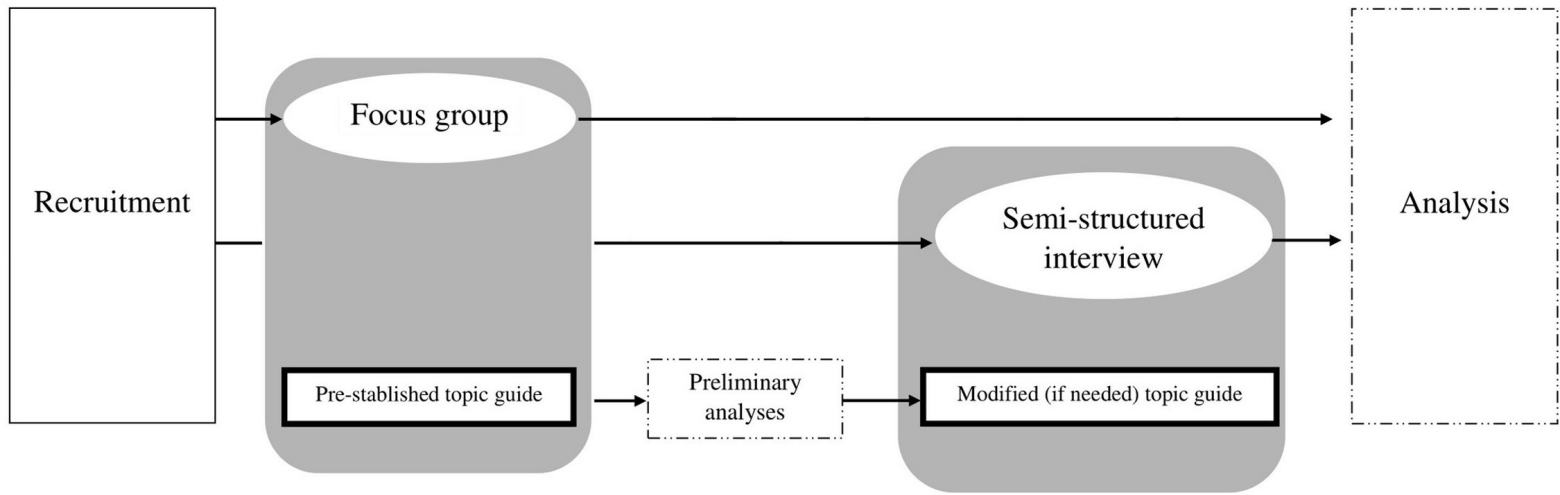
Study flow chart.

A structured script will be used to guide the dynamics of the focus group and semi-structured interviews, ensuring that all relevant topics are discussed (Table 1). Similarly, this script may be expanded or modified in response to the issues raised by the participants. The focus group data may be used to modify the topics to be discussed in the semi-structured interviews that will follow, if it is determined that relevant topics have emerged that were not previously included in the interview script (Fig 1). The focus group and each interview will last approximately an hour and a half to two hours.

**Table 1:** Topic guide for focus group and semi-structured interviews.

Broad topics	Specific discussion topics
Living with a pathology related to NKX2-1 gene mutation.	• Limitations in daily life
	• Quality of life
	• Well-being
Problems perceived in the care process.	• Diagnostic and treatment pathways
	• Follow-ups
	• Degree of satisfaction
Information needs.	• General information needs
	• Communication with professionals
	• Other sources of information (Internet or written documents)
	• Decision-making
	• Recommendations for other patients.

### Data analysis

The focus group and the semi-structured interviews will be transcribed *verbatim*. Transcripts will be analysed using the NVivo V.12 software package by QSR International [23]. The analysis of the transcripts will be based on Grounded Theory [24]. Thus, two researchers will independently analyse the transcript of the focus group and two semi-structured interviews. Each of them will develop their own topic proposal to categorise the elements identified by the participants. Each topic will consist of a collection of speeches describing the same situation or issue. In this way, an open coding will be developed. Open coding entails the coding of speeches on similar subjects under the same theme. The following step consists in reviewing each topic and grouping them together into larger units of meaning, a process known as axial coding. The final themes will emerge from an exchange of ideas between the two researchers. After establishing the final coding scheme, each researcher will conduct an independent analysis of the focus group and the four semi-structured interviews. Disagreements will be resolved through consensus.

### Data management

Focus group and interviews will be recorded in audio and video. They will be transcribed, analyzed and used to develop the ongoing CPG and accompanying patient information booklet. Also, the study findings will be published in an open access journal. Data will be stored under password and used for research purposes only. Considering the qualitative nature of the study and the sensitivity of the condition of potential participants, as they could be easily identified due to the rarity of this disease, raw data from the study will not be published in any repository.

### Ethics statement

This study protocol was approved by the Andalusian Regional Ministry of Health’s Ethics Coordinating Committee for Biomedical Research (Sevilla, Andalucía, Spain) on 29/03/2022. Each participant will be given an information sheet, an informed consent form and a confidentiality form. Both forms will require participant signatures. The collected data will be treated confidentially and anonymised throughout the entire study and its dissemination, for which each participant will be assigned an alphanumeric code.

## Discussion

In general, patients living with a disease can help clinicians and scientists understand the morbidities associated with the disease, and provide a context for the development of potential interventions [25]. This becomes even more necessary and specific in the case of rare diseases, where experiences with the healthcare process are different from those of common conditions [26]. Hence the need to know their perspectives and preferences when developing clinical decision support documents such as CPGs.

Focus on *NKX2-1-* related disorders, and as far as we are aware, this is the first study to investigate the values, preferences, and information needs of patients with *NKX2-1*-related disorders. The proposed study’s findings will contribute to the generation of useful knowledge that will be used to develop the ongoing Clinical Practice Guideline and accompanying patient information booklet, likewise, it will be published in international peer-reviewed journals and presented in conferences. It is further expected that given the focus on patient preference of this study, the results deriving from this study may have a positive impact on the implementation of CPG-derived recommendations and their acceptance by patients and caregivers, and also, improve the care they receive and their satisfaction with same and better informed shared clinical decision-making.

The major anticipated limitation of this study is that, while it will provide valuable insights into the perspectives of patients with *NKX2-1*-related disorders, the findings are unlikely to be generalizable to patients with other conditions. However, conducting this study and disseminating it may serve as a wake-up call of the need to integrate the patient perspective into CPGs, especially those addressing rare diseases.
